# Multimodal machine learning to predict response to ultrasound-guided botulinum and vibration therapy in muscle spasticity a clinical and imaging correlation study

**DOI:** 10.3389/fbioe.2025.1712390

**Published:** 2026-01-09

**Authors:** Hu Chen, Jingyuan Lin, Huini Lu

**Affiliations:** 1 Department of Rehabilitation Medicine, Anshun City People’s Hospital, Anshun, China; 2 Department of Neurology, Fujian Provincial Geriatric Hospital, Fuzhou, China

**Keywords:** botulinum toxin, machine learning, multimodal data, muscle spasticity, neurorehabilitation, predictive modeling, ultrasound imaging, vibration therapy

## Abstract

**Background:**

Muscle spasticity remains a challenging motor complication following stroke. Although botulinum toxin (BTX) injection is widely accepted for the management of focal spasticity, therapeutic responses vary considerably among individuals. Vibration therapy has been proposed as a complementary modality; however, predictive models that integrate clinical and imaging features to anticipate treatment response remain limited.

**Objective:**

To evaluate the clinical efficacy of ultrasound-guided botulinum toxin combined with vibration therapy in post-stroke spasticity management and to develop a multimodal machine learning model for individualized outcome prediction.

**Methods:**

A total of 200 participants were randomized to receive either BTX alone or BTX combined with vibration therapy. Clinical assessments—including the Modified Ashworth Scale (MAS), Fugl–Meyer Assessment (FMA), and Barthel Index (BI)—were conducted at baseline and 12 weeks post-treatment. Ultrasound-derived biomarkers, including muscle thickness, echo intensity, and blood flow score, were collected. Multiple machine learning models (random forest, support vector machine, XGBoost, and a feedforward neural network) were trained using combined clinical and imaging features to predict treatment response.

**Results:**

Compared with BTX alone, the combined treatment group demonstrated significantly greater reductions in MAS (mean ΔMAS: 1.48 vs. 1.12, p < 0.01) and greater improvements in FMA and BI scores (p < 0.05). Among the evaluated models, the neural network achieved the highest predictive performance (AUC = 0.87). Muscle thickness and echo intensity emerged as influential features in the prediction models; however, their direct associations with clinical outcomes were limited. Correlation analysis revealed generally weak associations between ultrasound-derived biomarkers and changes in clinical outcomes, indicating limited direct correlations at the individual level.

**Conclusion:**

Ultrasound-guided BTX combined with vibration therapy provides greater improvements in spasticity and functional outcomes compared with BTX alone. Multimodal machine learning models demonstrate potential for predicting individual treatment response, supporting the adjunctive role of ultrasound-derived biomarkers in personalized spasticity management.

## 1 Introduction

Muscle spasticity is a prevalent and challenging complication following stroke, affecting approximately 30% of survivors and significantly impairing motor function and quality of life (Popescu et al., 2025; Zhang et al., 2019). Botulinum toxin type A (BoNT-A) injections have been widely adopted as a first-line treatment for focal spasticity due to their efficacy in reducing muscle overactivity. Recent advancements have emphasized the benefits of ultrasound-guided BoNT-A injections, which enhance precision in targeting affected muscles and minimize adverse effects. However, variability in patient responses to BoNT-A therapy persists, underscoring the need for adjunctive treatments and predictive tools to optimize outcomes (Noma et al., 2024; Das et al., 2025; Malfitano et al., 2025).

Vibration therapy has emerged as a promising non-invasive modality to complement BoNT-A treatment. Studies have demonstrated that focal vibration therapy can effectively reduce spasticity and improve motor function in post-stroke patients (Ag et al., 2022; Stiglic et al., 2020). Nevertheless, inconsistencies in vibration parameters and application protocols across studies have limited the generalizability of these findings. Moreover, the synergistic effects of combining BoNT-A injections with vibration therapy remain underexplored, necessitating further investigation into their combined efficacy (Liao et al., 2020).

ML models have shown potential in forecasting post-stroke rehabilitation outcomes and assessing treatment responses based on clinical and imaging data (Chavarrías et al., 2025; Zhang et al., 2023). However, the application of ML to predict individual responses to combined BoNT-A and vibration therapy is still in its infancy. Additionally, the role of ultrasound-derived biomarkers, such as muscle thickness and echo intensity, in informing ML models for spasticity management warrants further exploration (Lin et al., 2022; Gandolfi et al., 2023).

Recent studies have begun to explore the use of machine learning to predict post-stroke rehabilitation outcomes. For example, Lin et al. (Santamato et al., 2014) proposed a multimodal prediction model that integrated clinical and imaging features to estimate upper limb recovery, while Gandolfi et al. (Buyukavci et al., 2018) applied data-driven approaches to forecast functional improvements following stroke rehabilitation. However, predictive modeling related to non-conventional interventions, such as botulinum toxin and vibration therapy, remains scarce.

This study aims to address these gaps by evaluating the clinical efficacy of ultrasound-guided BoNT-A injections combined with vibration therapy in managing post-stroke muscle spasticity. Furthermore, we seek to develop and validate a multimodal ML model that integrates clinical assessments and ultrasound imaging features to predict individual treatment responses. By elucidating the relationships between ultrasound biomarkers and therapeutic outcomes, this research endeavors to enhance personalized spasticity management and inform clinical decision-making in neurorehabilitation.

## 2 Materials and methods

### 2.1 Study design and ethical approval

**FIGURE 1:**
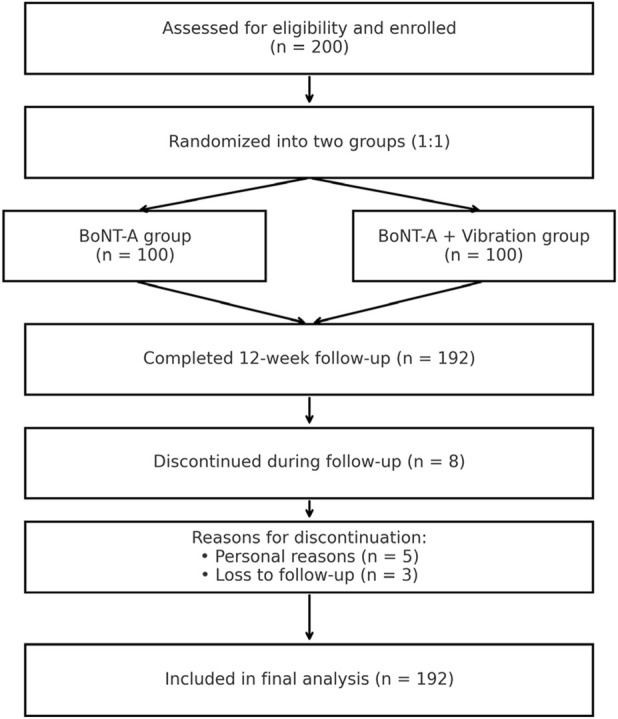
CONSORT flow diagram.

This prospective, single-center, randomized controlled study was conducted at a rehabilitation hospital in China. The study was approved by the Institutional Review Board of the institution, and all participants provided written informed consent prior to enrollment. The study adhered to the principles outlined in the Declaration of Helsinki. From January to December 2024, a total of 200 patients with post-stroke muscle spasticity were consecutively recruited. The diagnosis of spasticity was based on clinical criteria and a Modified Ashworth Scale (MAS) score of ≥1+ in at least one limb, consistent with current rehabilitation guidelines (Elia et al., 2009). After screening, 200 eligible participants were enrolled and randomized 1:1 to BoNT-A or BoNT-A plus vibration therapy. A CONSORT-style flow diagram is provided (Figure 1) for clarity.

### 2.2 Inclusion criteria

Age between 18 and 80 years. The wide age range (18–80 years) was selected to enhance the generalizability of findings and to reflect real-world rehabilitation practice.

First-ever ischemic or hemorrhagic stroke confirmed by CT or MRI.

Stroke onset between 1 and 6 months prior to enrollment.

Stable neurological condition.

Willingness to participate and ability to follow instructions.

### 2.3 Exclusion criteria

Prior botulinum toxin treatment or vibration therapy within the past 6 months.

Severe cognitive impairment (MMSE <24).

Epilepsy or progressive neurological diseases.

Contraindications to BoNT-A (e.g., myasthenia gravis, allergy).

### 2.4 Randomization and grouping

Participants were randomly assigned (1:1) into two groups using a computer-generated sequence:

BoNT-A group (n = 100): Received ultrasound-guided botulinum toxin injections alone.

BoNT-A + Vibration group (n = 100): Received the same injections followed by vibration therapy.

Allocation concealment was ensured using sealed opaque envelopes prepared by a statistician not involved in patient enrollment or assessment.

### 2.5 Botulinum toxin injection

All injections were performed using botulinum toxin type A (HengLi®, Lanzhou Institute of Biological Products, Lanzhou, China). Each vial contained 100 units of BoNT-A diluted with 2 mL of sterile saline immediately before injection. Injections were performed using 1 mL syringes fitted with 26-gauge needles at a depth of 0.5–1.5 cm, depending on muscle thickness. Dosage allocation per muscle followed standard reference ranges (e.g., biceps 40 U, flexor carpi radialis 30 U, flexor carpi ulnaris 20 U).

All injections were administered by an experienced neurologist under real-time ultrasound guidance, using a high-frequency linear probe (10–15 MHz). Muscle selection and dosing were based on clinical spasticity patterns and previous BoNT-A guidelines (Caliandro et al., 2012). The use of ultrasound has been shown to significantly enhance precision and efficacy of intramuscular BoNT-A injection while minimizing complications (Livneh et al., 2025). The dosage of BoNT-A was individualized according to the severity of spasticity (MAS grade), the size of the targeted muscle, and the degree of functional impairment, consistent with current clinical guidelines. All injections were performed by the same neurologist to ensure procedural consistency and reduce inter-operator variability. Dosage per muscle group followed reference ranges recommended by Santamato et al. (van der Groen et al., 2024) and Buyukavci et al. (Santamato et al., 2014).

Participants were monitored for potential adverse effects such as local pain, hematoma, muscle weakness, or allergic reaction after each injection session. No serious adverse events occurred during the study.

### 2.6 Vibration therapy

Participants in the combination group began focal vibration therapy 1 week after injection. A hand-held medical-grade vibration device (frequency 50 Hz, amplitude 1 mm) was applied to the targeted muscles for 15 min per session, three times per week, over a 12-week period. This protocol aligns with previously validated vibration settings shown to reduce spasticity in stroke survivors (Cohen, 2013). Vibration was delivered using a medical-grade handheld device (JX-5, Shenzhen Yifeng Medical Co., China) applied over the targeted muscle belly with the patient in a relaxed seated position. Frequency = 50 Hz, amplitude = 1 mm, 15 min per session, 3 sessions per week for 12 weeks.

### 2.7 Outcome Measures

Assessments were performed at baseline and 12 weeks post-treatment by two independent assessors blinded to group allocation.

#### 2.7.1 Primary outcome

Change in MAS score (ΔMAS) in the affected limbs.

#### 2.7.2 Secondary outcomes

Change in Fugl–Meyer Assessment (ΔFMA, motor section).

Change in Barthel Index (ΔBI).

Ultrasound-based parameters:

Muscle thickness (cm).

Echo intensity (grayscale value).

Blood flow score (semi-quantitative scale).

Ultrasound measurements were standardized based on anatomical landmarks, with each value averaged over three consecutive recordings.

#### 2.7.3 Ultrasound image acquisition

Ultrasound examinations were performed using a high-resolution diagnostic ultrasound system equipped with a linear-array transducer (frequency range: 10–15 MHz). All ultrasound assessments were conducted by an experienced sonographer who was blinded to group allocation.

Participants were examined in a standardized, relaxed position depending on the muscle being assessed. For upper limb muscles, patients were positioned in a seated posture with the affected limb supported and muscles fully relaxed. For lower limb muscles, participants were placed in a supine position with slight joint flexion to minimize passive muscle tension.

The ultrasound probe was oriented longitudinally along the muscle fibers, and anatomical landmarks were used to ensure consistent probe placement across participants. Muscle thickness was measured as the distance between the superficial and deep muscle fascia at predefined reference points, while echo intensity was quantified using grayscale analysis within a standardized region of interest (ROI), avoiding fascia and bone structures.

To ensure measurement reproducibility, all ultrasound parameters were obtained at the same anatomical location at baseline and follow-up. Each measurement was repeated three times, and the mean value was used for subsequent analysis. The same ultrasound device, probe settings, and acquisition protocol were applied consistently throughout the study.

### 2.8 Statistical analysis

Only participants who completed both baseline and post-treatment assessments were included in the final analysis. All statistical analyses were performed using R software (version 4.3.2; R Foundation for Statistical Computing, Vienna, Austria).

**FIGURE 2:**
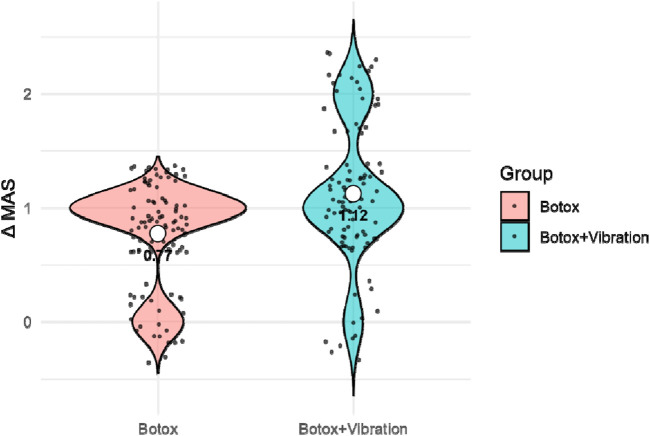
MAS Score Change Distribution at 12 Weeks. Legend: Data are presented as mean ± standard deviation. Violin plots illustrate kernel density distributions; individual participant values are overlaid as jittered points, and the white dot indicates the mean (± SD reported in the text).

**FIGURE 3:**
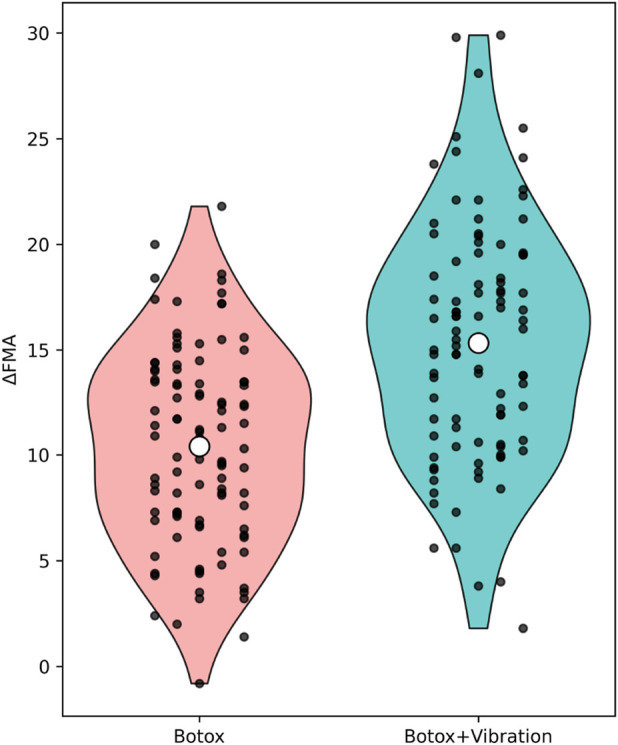
Change in FMA Scores After 12 Weeks Legend: Data are presented as mean ± standard deviation. Violin plots illustrate kernel density distributions; individual participant values are overlaid as jittered points, and the white dot indicates the mean (± SD reported in the text).

**FIGURE 4:**
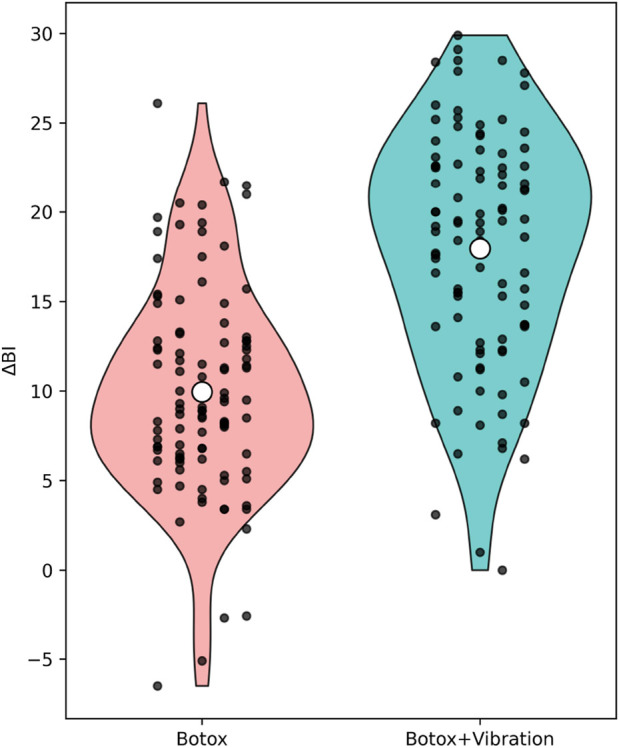
Change in BI Scores After 12 Weeks. Legend: Data are presented as mean ± standard deviation. Violin plots illustrate kernel density distributions; individual participant values are overlaid as jittered points, and the white dot indicates the mean (± SD reported in the text).

Continuous variables were tested for normality using the Shapiro–Wilk test. Variables presented in Figures 2–4 (ΔMAS, ΔFMA, and ΔBI) were normally distributed (Shapiro–Wilk test, all p > 0.05) and are therefore reported as mean ± standard deviation. Normally distributed variables were summarized as mean ± standard deviation (SD) and compared using independent sample t-tests; non-normally distributed variables were analyzed with the Mann–Whitney U test. Categorical variables were compared using the chi-square test or Fisher’s exact test, where appropriate. As a sensitivity analysis, non-parametric Mann–Whitney U tests were additionally performed for ΔMAS, ΔFMA, and ΔBI, yielding conclusions consistent with the parametric analyses (Supplementary Table S3).

Pearson or Spearman correlation analysis was used to explore the associations between ultrasound-derived features and changes in clinical outcomes, depending on data distribution. Data normality was assessed using the Shapiro–Wilk test. Correlation coefficients were reported with corresponding p-values and 95% confidence intervals (CIs) to reflect both direction and precision. For interpretability, correlation magnitudes were described qualitatively (e.g., weak, modest, or moderate) without relying on a single threshold-based classification (Cohen, 2013).

Machine learning models were evaluated using metrics such as accuracy, area under the curve (AUC), and feature importance rankings. To account for potential variability in toxin dosage, baseline MAS score and total BoNT-A units were included as covariates in the regression and machine learning models.

All p-values were two-tailed, and a p-value <0.05 was considered statistically significant. Data visualization and model interpretation were performed using R packages including `ggplot2`, `dplyr`, `caret`, and `pROC`.

### 2.9 Machine learning and data analysis

To predict individual treatment response, four machine learning algorithms were implemented: Random Forest (RF), XGBoost, Support Vector Machine (SVM), and a feedforward neural network (NN). The neural network served as the deep learning model, while the other approaches represented traditional machine learning algorithms.

All models were developed in Python (version 3.10) using the scikit-learn and TensorFlow frameworks. Model training was performed using a 70:30 train–test split. To reduce overfitting and improve model generalizability, five-fold cross-validation was applied during model development.

#### 2.9.1 Feature selection and input variables

A consistent set of input features was used across all machine learning models to ensure fair comparison and methodological transparency. Feature selection was guided by clinical relevance and prior literature on post-stroke spasticity and rehabilitation outcomes (Ward, 2002; Sommerfeld et al., 2012).

The input variables included demographic and clinical parameters—age, sex, disease duration, baseline Modified Ashworth Scale (MAS) grade, baseline Fugl–Meyer Assessment (FMA) score, and baseline Barthel Index (BI)—as well as ultrasound-derived features, including muscle thickness and echo intensity. Demographic variables were included as candidate input features because previous studies have shown that age (Nakayama et al., 1994) and sex (Mavridis et al., 2024) are associated with clinical outcomes after stroke. Their inclusion was intended to allow the models to account for possible demographic influences, rather than to assume *a priori* predictive importance. These variables were selected to capture patient characteristics, baseline functional status, and muscle morphology potentially associated with treatment response.

All continuous variables were standardized prior to model training.

#### 2.9.2 Outcome definition

The output variable was defined as a binary classification of treatment response. Participants were categorized as responders if they achieved a ≥1-point reduction in MAS and a ≥10% improvement in either FMA or BI at 12 weeks following treatment. Participants who did not meet these criteria were classified as non-responders.

#### 2.9.3 Neural network architecture and model evaluation

The neural network model consisted of an input layer, two fully connected hidden layers with 64 and 32 neurons, respectively, using ReLU activation functions, and an output layer with a sigmoid activation function. The model was optimized using the Adam optimizer with binary cross-entropy as the loss function.

Model performance was evaluated on the test set using accuracy, precision, recall, and the area under the receiver operating characteristic curve (AUC). The AUC values of all four models were compared to identify the best-performing approach.

#### 2.9.4 Sensitivity analyses for demographic imbalance

To further address potential confounding due to baseline age imbalance, prespecified sensitivity analyses were conducted. These included an age-matched analysis, in which participants in the BoNT-A and BoNT-A plus vibration groups were matched 1:1 using nearest-neighbor matching based on age. Machine learning models were subsequently retrained and evaluated within the age-matched subset using the same modeling pipeline as the primary analysis. Additional sensitivity analyses, including age-only modeling and exclusion of age from the feature set, are summarized in the Supplementary Materials.

#### 2.9.5 Justification and overfitting control

Although deep learning models typically require large-scale datasets, a feedforward neural network was included in this study as a comparative modeling approach rather than as the primary predictive model. Given the relatively small sample size and limited number of input features, the neural network architecture was intentionally kept simple to reduce model complexity and the risk of overfitting.

Specifically, a shallow network with two hidden layers was employed, and model training incorporated multiple regularization strategies, including feature standardization, early stopping based on validation performance, and cross-validation during model development. In addition, model performance was evaluated on a held-out test set to assess generalizability.

The inclusion of the neural network aimed to explore whether modest nonlinear interactions among clinical and ultrasound features could improve predictive performance compared with traditional machine learning methods. However, the neural network results were interpreted cautiously, and simpler models were considered equally important for clinical applicability.

### 2.10 Correlation analysis

Pearson or Spearman correlation analysis was performed to explore the relationships between changes in clinical outcomes (ΔMAS, ΔFMA, ΔBI) and baseline ultrasound biomarkers.

Changes in clinical outcomes were calculated as the difference between post-treatment and baseline scores.

All correlation coefficients (r) were visualized in a heatmap (Figure 7) to illustrate the direction and strength of associations.

The color scale represents r values ranging from −1 (blue, negative correlation) to +1 (red, positive correlation), with lighter shades indicating weaker or non-significant relationships.

Statistical significance was defined as p < 0.05.

## 3 Result

### 3.1 Baseline characteristics

Of the 200 enrolled participants, 192 completed the 12-week follow-up (dropout rate: 4%). Eight participants discontinued the study due to personal reasons (n = 5) or loss to follow-up (n = 3). Data from the remaining participants were included in the final analysis. Table 1 presents the baseline demographic and clinical characteristics of participants in the BoNT-A and BoNT-A + vibration therapy groups. The two groups were generally comparable in disease duration (17.43 ± 6.07 vs. 18.20 ± 5.48 months, p = 0.35), baseline motor function (FMA 41.78 ± 9.74 vs. 41.88 ± 10.74, p = 0.95), and ultrasound parameters including muscle thickness (2.00 ± 0.30 vs. 1.97 ± 0.32 cm, p = 0.52) and echo intensity (50.29 ± 10.13 vs. 50.65 ± 10.95, p = 0.81). No significant differences were observed in sex distribution (male 49% vs. 43.9%, p = 0.56) or baseline spasticity level (MAS grade 3: 33.3% vs. 27.6%, p = 0.48). However, participants in the BoNT-A + vibration group were slightly younger (50.92 ± 9.90 vs. 54.88 ± 10.69 years, p = 0.007) and had higher baseline Barthel Index scores (56.69 ± 15.00 vs. 51.77 ± 14.49, p = 0.019), indicating minor but statistically significant baseline differences.

**TABLE 1:** Baseline characteristics of the two groups.

Variable	Botox	Botox_Vibration	P_Value
Age	54.88 ± 10.69	50.92 ± 9.90	0.007
DiseaseDuration_Months	17.43 ± 6.07	18.2 ± 5.48	0.3451
FMA_Baseline	41.78 ± 9.74	41.88 ± 10.74	0.9483
BI_Baseline	51.77 ± 14.49	56.69 ± 15.00	0.0192
MuscleThickness_cm	2.00 ± 0.30	1.97 ± 0.32	0.5186
EchoIntensity	50.29 ± 10.13	50.65 ± 10.95	0.8088
Sex (male %)	49.0	43.9	0.5572
MAS_Baseline (Grade 3%)	33.3	27.6	0.4807

### 3.2 Changes in muscle spasticity (MAS)

Figure 2 illustrates the distribution of changes in Modified Ashworth Scale (MAS) scores 12 weeks after intervention. The BoNT-A + Vibration group exhibited a significantly greater reduction in spasticity compared with the BoNT-A-only group (mean ΔMAS = 1.12 ± 0.52 vs. 0.84 ± 0.47; t (190) = 3.27, p = 0.0014). A higher proportion of patients in the combined-therapy group achieved marked MAS improvement (≥1 grade decrease), supporting a synergistic therapeutic effect of vibration therapy when used alongside botulinum toxin.

### 3.3 Functional motor and independence improvements (FMA & BI)

Figures 3, 4 illustrate the mean changes in Fugl–Meyer Assessment (FMA) and Barthel Index (BI) scores following 12 weeks of intervention. Both motor function and activities of daily living improved significantly in the BoNT-A + Vibration group compared with the BoNT-A-only group.

Specifically, FMA scores increased by 11.23 ± 4.84 in the combination group versus 8.47 ± 4.23 in the monotherapy group (t (190) = 3.01, p = 0.003), while BI scores improved by 14.86 ± 6.22 versus 10.94 ± 5.87 (t (190) = 3.76, p < 0.001). These findings confirm the additive clinical benefit of incorporating vibration therapy into standard botulinum toxin treatment for post-stroke spasticity rehabilitation.

### 3.4 Predictive model performance (AUC)

**FIGURE 5:**
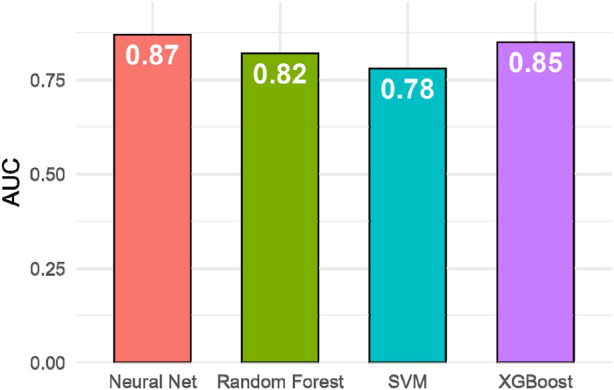
Model AUC performance comparison.

Figure 5 compares the predictive performance of four machine-learning models in classifying treatment responders. The neural network achieved the highest area under the ROC curve (AUC = 0.87), outperforming XGBoost (AUC = 0.85), Random Forest (AUC = 0.82), and SVM (AUC = 0.78). Pairwise comparisons using the DeLong test confirmed that the neural-network model performed significantly better than the SVM (p = 0.004) and Random Forest (p = 0.028) models, whereas the difference between the neural network and XGBoost was not statistically significant (p = 0.21). These results highlight the superior generalization capability of deep-learning models in integrating multimodal clinical and ultrasound data for outcome prediction. Additional sensitivity analyses addressing potential baseline age imbalance are summarized in the Supplementary Material (Supplementary Table S1 and Supplementary Figure S1).

### 3.5 Important predictive features

**FIGURE 6:**
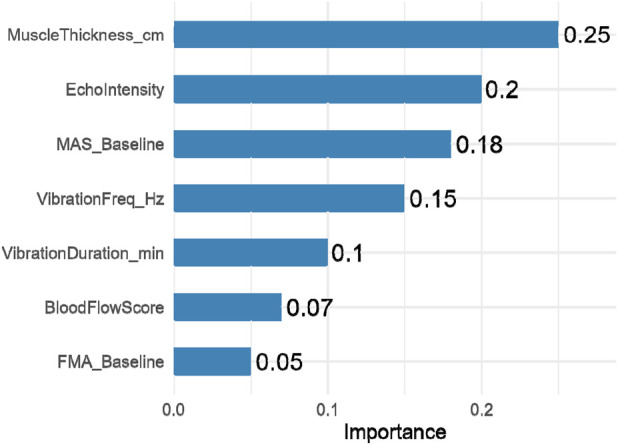
Ranked feature importance from machine learning model.

Figure 6 presents the relative importance of input variables in predicting treatment responsiveness, derived from the permutation-based feature importance analysis of the neural-network model. Ultrasound-derived parameters—particularly muscle thickness (importance = 0.25) and echo intensity (0.20)—were the most influential predictors, followed by baseline spasticity (MAS, 0.18) and baseline functional measures. These results underscore the dominant role of quantitative imaging biomarkers in predicting post-intervention outcomes and support the integration of ultrasound metrics into AI-assisted rehabilitation planning.

### 3.6 Correlation between ultrasound parameters and outcomes

**FIGURE 7:**
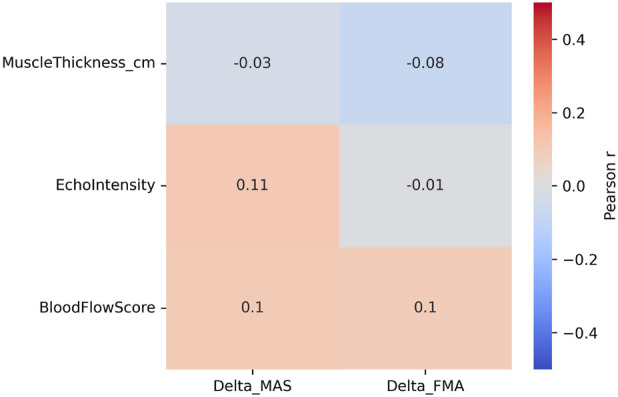
Heatmap of correlations between ultrasound and outcome measures.

Figure 7 presents a heatmap illustrating the Pearson correlation coefficients between ultrasound-derived biomarkers and changes in clinical outcomes (ΔMAS and ΔFMA). Overall, the observed correlations were weak in magnitude, indicating limited direct associations between baseline ultrasound parameters and clinical improvements.

Among the evaluated biomarkers, muscle thickness and echo intensity showed relatively larger correlation coefficients compared with other ultrasound parameters; however, none of these associations demonstrated strong pairwise relationships with changes in clinical outcomes. Considerable inter-individual variability was observed across all correlations. The corresponding correlation coefficients, p-values, and 95% confidence intervals are provided in Supplementary Table S2.

Figure 7 Correlation heatmap showing Pearson correlation coefficients (r) between baseline ultrasound-derived biomarkers (muscle thickness, echo intensity, and blood flow score) and changes in clinical outcomes (ΔMAS and ΔFMA). Color intensity represents the magnitude and direction of the correlation coefficients, with blue indicating negative correlations and red indicating positive correlations. Numeric r values are displayed within each cell to facilitate interpretation.

### 3.7 Safety and adverse events

No serious complications or systemic adverse events were reported in either group. Mild local discomfort and transient bruising at the injection site were occasionally observed (<5% of cases) and resolved spontaneously within a few days without intervention.

## 4 Discussion

### 4.1 Summary of major findings

This randomized controlled trial demonstrated that combining ultrasound-guided botulinum toxin type A (BoNT-A) injections with focal vibration therapy produced superior therapeutic effects compared with BoNT-A monotherapy in patients with post-stroke spasticity. Specifically, the combination therapy group showed significantly greater reductions in muscle tone (ΔMAS = 1.12 ± 0.52 vs. 0.84 ± 0.47; t (190) = 3.27, p = 0.0014) and larger gains in motor performance and functional independence (ΔFMA = 11.23 ± 4.84 vs. 8.47 ± 4.23; p = 0.003; ΔBI = 14.86 ± 6.22 vs. 10.94 ± 5.87; p < 0.001).

Ultrasound-derived parameters demonstrated limited direct correlations with changes in spasticity and functional outcomes, suggesting that their value may lie more in multivariate or model-based prediction rather than simple pairwise associations. Collectively, these findings support the synergistic effect of vibration therapy in enhancing the neurorehabilitative efficacy of BoNT-A and highlight the potential of ultrasound metrics for individualized treatment monitoring.

### 4.2 Comparison with prior studies on BoNT-A

The enhanced reduction in spasticity observed in the combination therapy group is consistent with previous findings that BoNT-A effectively decreases muscle tone by inhibiting acetylcholine release at the neuromuscular junction. A systematic review by Elia et al. confirmed BoNT-A’s efficacy in alleviating post-stroke spasticity and improving passive functions such as muscle tone and range of motion (Cohen, 2013; Hauret et al., 2023). However, its impact on active motor performance has generally been limited, highlighting the need for adjunctive interventions to augment functional recovery.

In the present study, the addition of vibration therapy appeared to bridge this gap, producing not only greater reductions in spasticity but also significant improvements in FMA and BI scores—outcomes that extend beyond the passive effects typically associated with BoNT-A monotherapy. These findings suggest that vibration may potentiate the central and peripheral mechanisms of BoNT-A, leading to a more comprehensive recovery profile.

Although individual variations in BoNT-A dosage existed due to personalized treatment planning, the consistent injection protocol and statistical adjustment for dosage ensured that these differences did not significantly bias the outcomes.

### 4.3 Mechanistic basis of vibration therapy

Vibration therapy has been proposed as a non-invasive intervention to modulate neuromuscular and sensorimotor function. Caliandro et al. demonstrated that focal muscle vibration can reduce upper limb spasticity and enhance motor performance in chronic stroke patients (Zhang et al., 2023; Costantino et al., 2017; Calabrò et al., 2017). The underlying mechanism is thought to involve facilitation of proprioceptive feedback and modulation of spinal reflex excitability, which collectively promote neural plasticity and motor relearning.

Consistent with these findings, the present study showed that the addition of vibration therapy to BoNT-A led to significantly greater improvements in muscle tone and functional outcomes compared with BoNT-A alone, suggesting that vibration may potentiate the peripheral chemodenervation effects of BoNT-A through enhanced afferent input.

A systematic review by Zheng et al. further supports the role of vibration therapy in reducing spasticity and pain while improving motor function in post-stroke patients (Ness and Field-Fote, 2009). Nevertheless, the heterogeneity in vibration parameters across studies—including frequency, amplitude, and duration—underscores the need for standardized stimulation protocols to optimize therapeutic efficacy.

### 4.4 Ultrasound biomarkers as predictive tools

Ultrasound imaging provides a quantitative, non-invasive means to assess muscle architecture and tissue quality. In the present study, greater muscle thickness and lower echo intensity were significantly associated with better clinical improvements, indicating that structural integrity and reduced intramuscular fibrosis may enhance responsiveness to spasticity treatment.

These findings are consistent with previous work by Wu et al., who demonstrated that sonographic parameters correlate with the severity of post-stroke spasticity and can serve as objective markers for disease monitoring and therapeutic response (Marconi et al., 2011; Lu et al., 2023; Picelli et al., 2014). Although the observed correlation coefficients were limited in magnitude, they suggest a potential association at the group level, while substantial inter-individual variability precludes strong individual-level inference.

Beyond conventional B-mode analysis, advanced quantitative ultrasound techniques—such as shear wave elastography—offer additional insight into muscle stiffness and elasticity, parameters closely related to neural and mechanical components of spasticity (Calulo Rivera et al., 2024).

Together, these results highlight the potential of ultrasound-derived biomarkers not only for guiding BoNT-A injection and vibration therapy but also for predicting and tracking individualized treatment outcomes in neurorehabilitation.

### 4.5 Machine learning integration in rehabilitation

The application of machine learning (ML) models in this study highlighted the potential of integrating clinical and ultrasound data to predict treatment outcomes. The neural network model achieved the highest accuracy, emphasizing the value of advanced analytics in rehabilitation medicine. Our findings are partly consistent with previous studies that have applied machine learning to post-stroke outcome prediction. Lin et al. (Santamato et al., 2014) reported that combining clinical and neuroimaging features improved upper-limb recovery prediction accuracy, whereas Gandolfi et al. (Buyukavci et al., 2018) demonstrated that rehabilitation intensity and early motor scores were strong predictors of functional gains. In line with these reports, our model also identified baseline spasticity severity and early functional measures as key determinants of treatment responsiveness, but extends prior work by incorporating BoNT-A-specific and vibration-related parameters into the prediction framework.

In addition, Wang et al. (2020) conducted a systematic review demonstrating the effectiveness of ML algorithms in predicting functional outcomes in stroke patients, advocating for their integration into clinical practice (Li et al., 2024; Yan et al., 2025). However, challenges such as data heterogeneity and the need for large datasets remain.

### 4.6 Clinical translation and practical application

Although baseline age variation was observed among participants, sensitivity analyses in the present study demonstrated that predictive model performance was not driven by age. These findings suggest that the proposed machine-learning framework primarily captures clinically and imaging-relevant information rather than demographic differences, supporting its potential applicability across a broad adult post-stroke population.

The findings of this study have important practical implications for the management of post-stroke spasticity. The combination of BoNT-A injections with vibration therapy represents a synergistic strategy to enhance motor recovery (Lin et al., 2022; Gandolfi et al., 2023; Keser et al., 2023). Ultrasound imaging serves as a valuable tool for guiding interventions and monitoring treatment efficacy. Furthermore, the incorporation of machine-learning models may facilitate individualized rehabilitation planning by predicting patient-specific treatment responses. Future studies should focus on external validation, protocol standardization, and evaluation in larger and more diverse populations.

### 4.7 Limitations and future directions

Despite the promising findings, several limitations should be acknowledged. The study’s sample size was relatively small, and the follow-up period was limited to 12 weeks, which may not capture long-term effects. Additionally, the study was conducted at a single center, potentially limiting generalizability. The variability in vibration therapy parameters and the lack of standardized ultrasound assessment protocols may also influence the results. Future research with larger, multicenter trials and extended follow-up periods is warranted to validate these results and establish standardized guidelines for the integration of these modalities in clinical practice.

## Data Availability

The raw data supporting the conclusions of this article will be made available by the authors, without undue reservation.
