# Supplementary figures and images for: Multimodal machine learning to predict response to ultrasound-guided botulinum and vibration therapy in muscle spasticity a clinical and imaging correlation study
Source: Front Bioeng Biotechnol. 2026 Jan 9;13:1712390. doi: 10.3389/fbioe.2025.1712390 (PMC12827723; doi:10.3389/fbioe.2025.1712390)

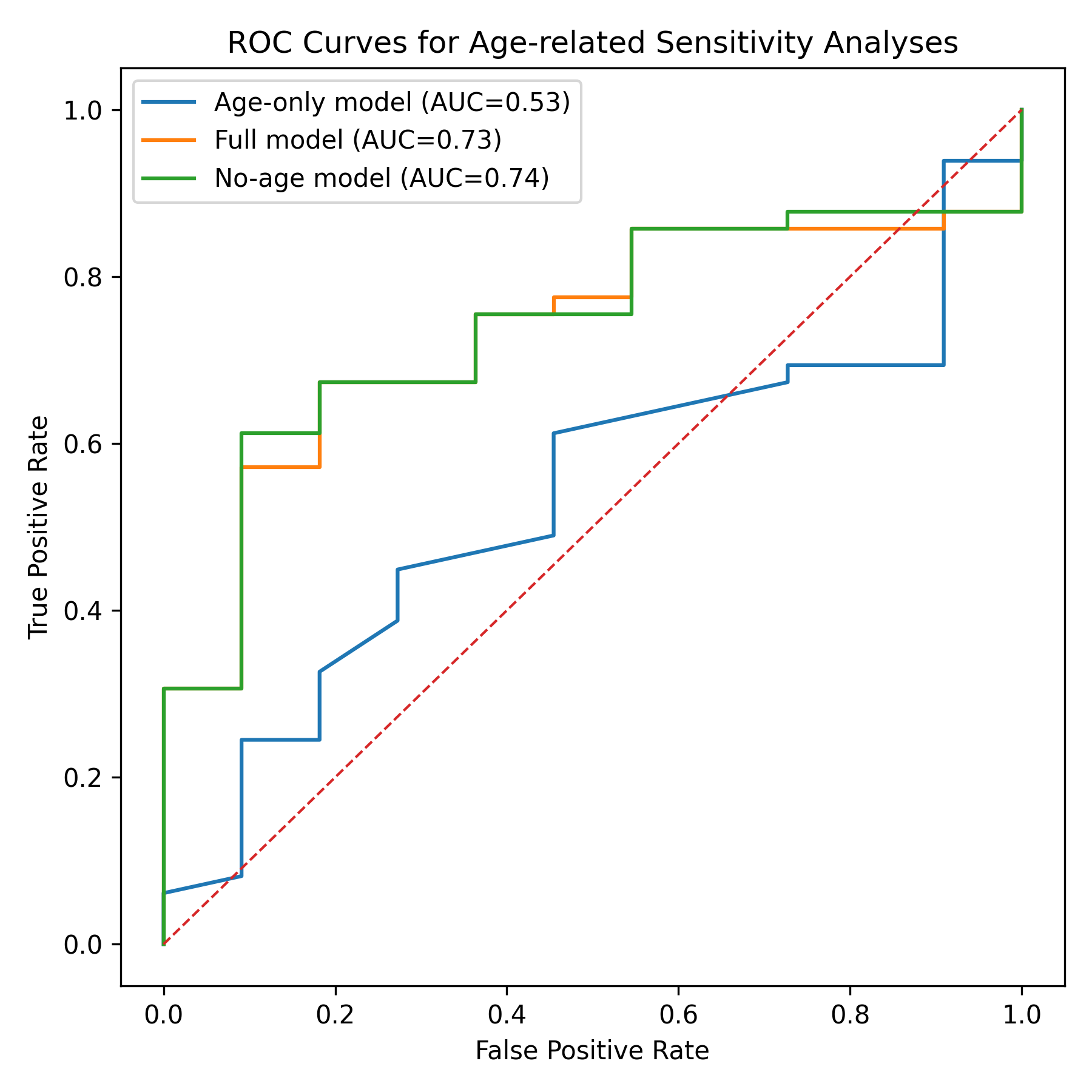

Supplement: Supplementary file 4 [file Image1.png]
